# Risk of dialysis in patients receiving intravitreal anti–vascular endothelial growth factor treatment: a population-based cohort study

**DOI:** 10.18632/aging.204133

**Published:** 2022-06-20

**Authors:** Shun-Fa Yang, Yu-Chen Su, Chen-Chee Lim, Jing-Yang Huang, Sheng-Min Hsu, Li-Wha Wu, Yi-Sheng Chang, Jia-Horung Hung

**Affiliations:** 1 Institute of Medicine, Chung Shan Medical University, Taichung, Taiwan; 2 Department of Medical Research, Chung Shan Medical University Hospital, Taichung, Taiwan; 3 Department of Ophthalmology, National Cheng Kung University Hospital, College of Medicine, National Cheng Kung University, Tainan, Taiwan; 4 Institute of Molecular Medicine, College of Medicine, National Cheng Kung University, Tainan, Taiwan; 5 Department of Laboratory Science and Technology, Kaohsiung Medical University, Kaohsiung, Taiwan; 6 Institute of Clinical Medicine, College of Medicine, National Cheng Kung University, Tainan, Taiwan

**Keywords:** population-based cohort study, intravitreal anti-vascular endothelial growth factor, aflibercept, ranibizumab, dialysis

## Abstract

We utilized the Longitudinal Health Insurance Database which was stemmed from the Taiwan's National Health Insurance Research Database to conduct a retrospective cohort study investigating the risk of becoming dialysis dependent after receiving intravitreal anti-vascular endothelial growth factor (VEGF) agents for retinal diseases. Patients newly receiving intravitreal ranibizumab or aflibercept from 2000 to 2017 for age-related macular degeneration, polypoidal choroidal vasculopathy, diabetic macular edema, retinal vein occlusions, or myopic choroid neovascularization were included as the study group, and patients with same retinal diseases but did not receive intravitreal anti-VEGFs served as controls extracted by age- and sex-matched (1:4) and further propensity score matching (PSM). Cox proportional hazards models were used to estimate hazard ratios (HR) and 95% confidence intervals (CI) for the risk of dialysis. A cohort of 2447 anti-VEGF users and 2447 controls by PSM were evaluated. Higher dialysis risks were observed among patients newly receiving anti-VEGF agents compared to controls (adjusted HR: 1.849; 95% CI: 1.378–2.482) in the PSM cohort. For subgroup analysis, patients newly receiving anti-VEGF treatment for diabetic macular edema had significant risk (adjusted HR: 1.834; 95% CI: 1.448–2.324) of becoming dialysis-dependent, while patients in other subgroups demonstrated similar risks as the controls. In conclusion, intravitreal anti-VEGF agents might increase the risk of becoming dialysis-dependent, especially in patients who are treated for diabetic macular edema.

## INTRODUCTION

End-stage renal disease (ESRD) is a dialysis-dependent status associated with a significant socioeconomic burden, and increasing age serves as a risk factor for mortality after dialysis initiation [1]. Every year, around 2.6 million people received renal replacement therapy due to ESRD worldwide [2], and Taiwan reportedly has a high prevalence of chronic kidney disease (15.46%) [3], along with the highest prevalence (0.33%) of treated ESRD in the world [4]. Among the etiologies of renal injury, drug-induced renal disorders play a major role, accounting for 18–27% of renal injuries [5].

Among the wide varieties of drugs that pose a threat to renal function, anti-vascular endothelial growth factor (VEGF) medications are often overlooked. Anti-VEGFs are widely used to prevent vascular proliferation in tumors and retinal diseases [6]. Anti-VEGF is extensively applied in the field of ophthalmology for indications that include exudative age-related macular degeneration (AMD), diabetic macular edema (DME), polypoidal choroidal vasculopathy (PCV), retinal vein occlusions (RVO), myopic choroid neovascularization (mCNV) and retinopathy of prematurity [7–9].

As the number of intravitreal anti-VEGF treatments has expanded in recent years [10–12], ophthalmologists are becoming aware of the associated adverse effects. Previous reports showed that hypertension is one of the adverse effects [13, 14], and risks of mortality and cerebrovascular accidents were found to be elevated in patients with poorer overall conditions [15, 16].

*In vitro* and animal studies have demonstrated that intravitreal anti-VEGF upregulates the inflammation in the kidney and retina [17], but current real-world evidence regarding renal complications after intravitreal anti-VEGF treatments remains scarce, and only case reports have demonstrated a possible correlation between intravitreal anti-VEGFs and kidney injuries [18–24]. Hence, by utilizing the National Health Insurance Research Database (NHIRD), a nationwide population-based dataset in Taiwan, a retrospective cohort study was conducted to investigate the risk of becoming dialysis-dependent after the administration of intravitreal anti-VEGFs. We hypothesized that the use of intravitreal anti-VEGFs was associated with increased risk of becoming dialysis dependent, compared with the control group.

## RESULTS

### Baseline characteristics of the study cohort

**Figure 1:**
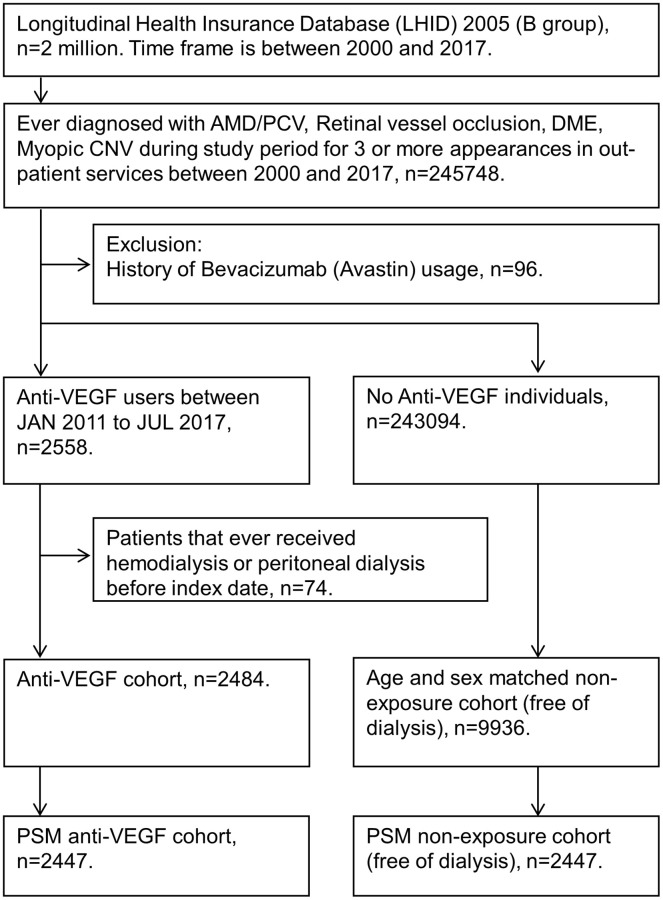
**Flow diagram showing study participant selection.** For patients who received intravitreal anti-vascular endothelial growth factor (VEGF) treatment, the index date was the day of the first intravitreal anti-vascular endothelial growth factor injection. For patients who did not receive intravitreal anti-vascular endothelial growth factor treatment, the index date was nested with the paired anti-VEGF patients. All study participants were at risk on the index date. Abbreviations: AMD: age-related macular degeneration; B group: 2005 Longitudinal Health Insurance Databases; CNV: choroidal neovascularization; DME: diabetic macular edema; PCV: polypoidal choroidal vasculopathy; PSM: propensity Score Matching; VEGF: vascular endothelial growth factor.

A total of 2484 subjects who received intravitreal injections of anti-VEGF were included in the study group, and another 9936 subjects without anti-VEGF matched by age and sex served as the control group. In addition, 2447 patients in the anti-VEGF group were matched with 2447 patients that did not receive anti-VEGF therapy using propensity score matching (PSM) (Figure 1). The differences in the baseline characteristics between the anti-VEGF and control groups were summarized in Table 1. In the cohort matched by age and sex, the patients in the anti-VEGF group were significantly more likely to have diabetes mellitus. After PSM, indications for anti-VEGF injection and comorbidities were similarly distributed in the two groups. In both cohorts (age- and sex-matched and PSM), most indications for anti-VEGF were AMD/PCV (47%) and DME (42%), and approximately 75% of anti-VEGF-treated patients received ranibizumab treatment.

**Table 1:** Baseline characteristics.

**Variable**	**Age-and sex-matched**			**PSM^a^**		
	**No anti-VEGF**	**Anti-VEGF**	**ASD^b^**	**No anti-VEGF**	**Anti-VEGF**	**ASD**
	***N* = 9936**	***N* = 2484**		***N* = 2447**	***N* = 2447**	
Year of index			0.0000			0.0231
2011–2013	2440 (24.56%)	610 (24.56%)		616 (25.17%)	602 (24.60%)	
2014–2015	3728 (37.52%)	932 (37.52%)		907 (37.07%)	918 (37.52%)	
2016–2017	3768 (37.92%)	942 (37.92%)		924 (37.76%)	927 (37.88%)	
Sex			0.0000			0.0117
Male	5948 (59.86%)	1487 (59.86%)		1479 (60.44%)	1465 (59.87%)	
Female	3988 (40.14%)	997 (40.14%)		968 (39.56%)	982 (40.13%)	
Age at index			0.0000			0.0277
20–40	185 (1.86%)	46 (1.85%)		24 (0.98%)	32 (1.31%)	
40–60	2166 (21.80%)	537 (21.62%)		511 (20.88%)	520 (21.25%)	
60–80	5979 (60.18%)	1501 (60.43%)		1514 (61.87%)	1495 (61.10%)	
80–100	1606 (16.16%)	400 (16.10%)		398 (16.26%)	400 (16.35%)	
Indication			0.4971			0.0000
AMD/PCV	3011 (30.30%)	1176 (47.34%)		1161 (47.45%)	1151 (47.04%)	
RVO	687 (6.91%)	129 (5.19%)		120 (4.90%)	129 (5.27%)	
DME	4245 (42.72%)	1052 (42.35%)		1043 (42.62%)	1040 (42.50%)	
Myopic CNV	1993 (20.06%)	127 (5.11%)		123 (5.03%)	127 (5.19%)	
Urbanization			0.0226			0.0751
Urban	6211 (62.51%)	1545 (62.20%)		1555 (63.55%)	1523 (62.24%)	
Sub-urban	2808 (28.26%)	719 (28.95%)		699 (28.57%)	709 (28.97%)	
Rural	917 (9.23%)	220 (8.86%)		193 (7.89%)	215 (8.79%)	
Insured unit type			0.0937			0.0661
Government	819 (8.24%)	177 (7.13%)		166 (6.78%)	176 (7.19%)	
Privately held company	5105 (51.38%)	1315 (52.94%)		1323 (54.07%)	1288 (52.64%)	
Agricultural organizations	1873 (18.85%)	470 (18.92%)		450 (18.39%)	467 (19.08%)	
Low-income	59 (0.59%)	15 (0.60%)		11 (0.45%)	14 (0.57%)	
Non-labor force	1910 (19.22%)	471 (18.96%)		467 (19.08%)	466 (19.04%)	
Others	170 (1.71%)	36 (1.45%)		30 (1.23%)	36 (1.47%)	
Marital status			0.0441			0.0643
Single	670 (6.74%)	168 (6.76%)		132 (5.39%)	151 (6.17%)	
Married	7957 (80.08%)	1964 (79.07%)		2013 (82.26%)	1947 (79.57%)	
Divorced	545 (5.49%)	150 (6.04%)		125 (5.11%)	147 (6.01%)	
Spouse deceased	764 (7.69%)	202 (8.13%)		177 (7.23%)	202 (8.26%)	
Education			0.1590			0.0519
≤9 years	4592 (46.22%)	1122 (45.17%)		1143 (46.71%)	1121 (45.81%)	
10–12 years	1381 (13.90%)	413 (16.63%)		411 (16.80%)	402 (16.43%)	
13–15 years	2890 (29.09%)	777 (31.28%)		737 (30.12%)	754 (30.81%)	
≥15 years	1073 (10.80%)	172 (6.92%)		156 (6.38%)	170 (6.95%)	
Co-morbidities						
Hypertension	5370 (54.05%)	1430 (57.57%)	0.0710	1398 (57.13%)	1407 (57.50%)	0.0074
Diabetes mellitus	5084 (51.17%)	1397 (56.24%)	0.1019	1371 (56.03%)	1367 (55.86%)	0.0033
IHD	744 (7.49%)	173 (6.96%)	0.0202	159 (6.50%)	172 (7.03%)	0.0212
Hyperlipidemia	3516 (35.39%)	953 (38.37%)	0.0618	927 (37.88%)	931 (38.05%)	0.0034
CHF	462 (4.65%)	108 (4.35%)	0.0146	95 (3.88%)	108 (4.41%)	0.0266
Rheumatic disease	103 (1.04%)	21 (0.85%)	0.0198	13 (0.53%)	21 (0.86%)	0.0394
Kidney disease	1268 (12.76%)	361 (14.53%)	0.0516	323 (13.20%)	348 (14.22%)	0.0297
CKD	580 (5.84%)	171 (6.88%)	0.0429	169 (6.91%)	165 (6.74%)	0.0065
Type of Anti-VEGF						
Ranibizumab	0 (0.00%)	1874 (75.44%)	–	0 (0.00%)	1845 (75.40%)	–
Aflibercept	0 (0.00%)	610 (24.56%)	–	0 (0.00%)	602 (24.60%)	–

Abbreviations: AMD: Age-related macular degeneration; CHF: Congestive heart failure; CKD: Chronic kidney disease; CNV: Choroidal neovascularization; DME: Diabetic macular edema; IHD: Ischemic heart diseases; PCV: Polypoidal choroidal vasculopathy; RVO: Branch retinal vein occlusion or central retinal vein occlusion; VEGF: Vascular endothelial growth factor. ^a^Propensity score matching (PSM) was done by matching: Year of index, Sex, Age at index, Indication, Urbanization, Insured unit type, Marital status, Education level, Co-morbidities (including Hypertension, Diabetes mellitus, Ischemic heart diseases, Hyperlipidemia, Congestive heart failure, and Rheumatic disease). ^b^Absolute standardized difference (ASD): >0.1 implies a meaningful imbalance between the groups.

### Comparison of the incidence rates and cumulative risk of dialysis between anti-VEGF-administered patients and patients without anti-VEGF treatment

**Figure 2:**
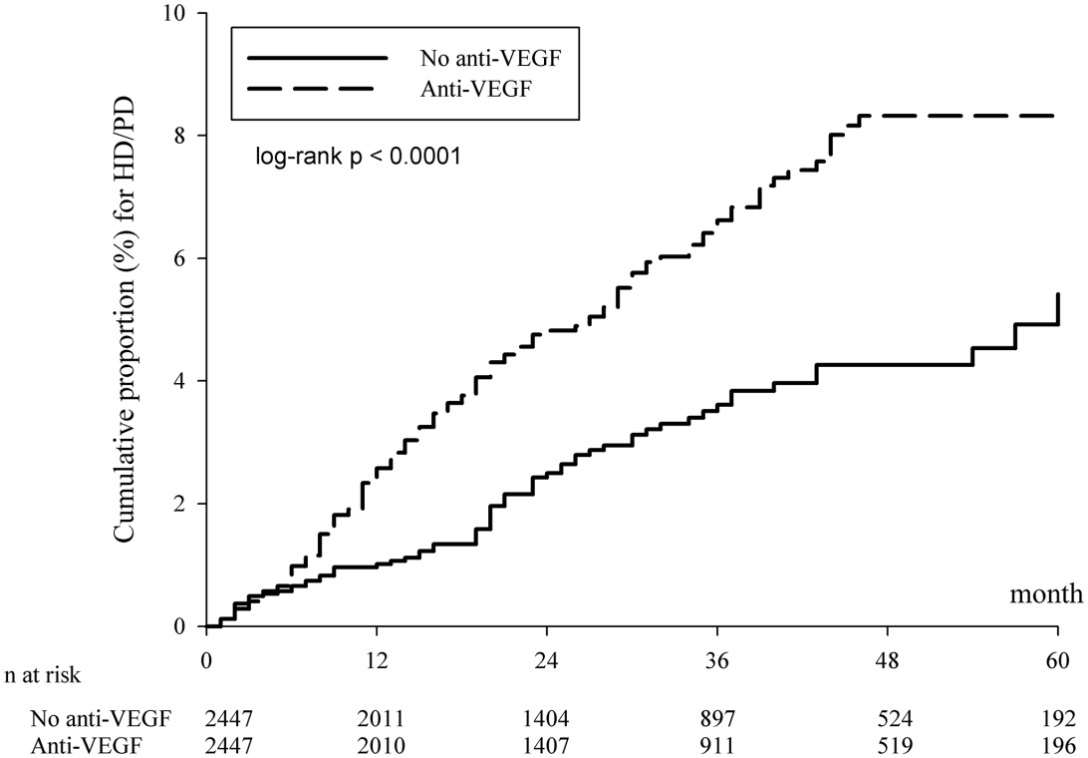
**Kaplan-Meier curves of the cumulative proportion for hemodialysis/peritoneal dialysis in the propensity score matched cohort.** Abbreviations: HD: hemodialysis; PD: peritoneal dialysis; VEGF: vascular endothelial growth factor.

In the PSM cohort, the incidence rate of dialysis per 10,000 person-months was 17.58 (95% CI: 14.82–20.85) in the anti-VEGF group, which was significantly higher than that in the group without anti-VEGF treatment: 9.37 (95% CI: 7.41–11.84) (Table 2). There was a significant difference in the cumulative risk of dialysis between the cohorts over the entire Kaplan–Meier curve (*p* < 0.0001, log rank test; Figure 2), and the adjusted hazard ratio was 1.849 (95% CI: 1.378–2.482) in the PSM cohort (Table 2). In addition, hypertension, diabetes, and congestive heart failure were also associated with the increased risk of dialysis (Supplementary Table 1). Moreover, the results also revealed a higher risk of dialysis in the anti-VEGF group when the endpoints of hemodialysis (HD) and peritoneal dialysis (PD) were analyzed separately (Table 2).

**Table 2:** Incidence and risk of hemodialysis or peritoneal dialysis among study groups.

	**Age-and sex-matched**		**PSM^a^**	
	**No anti-VEGF *N* = 9936**	**Anti-VEGF *N* = 2484**	**No anti-VEGF *N* = 2447**	**Anti-VEGF *N* = 2447**
HD or PD				
Follow up person months	302467	76142	74741	75080
New case	281	137	70	132
Incidence rate^b^ (95% C.I.)	9.29 (8.26–10.44)	17.99 (15.22–21.27)	9.37 (7.41–11.84)	17.58 (14.82–20.85)
Adjusted Hazard ratio (95% C.I.)	Reference	1.680 (1.358–2.078)	Reference	1.849 (1.378–2.482)
Hemodialysis				
Follow up person months	302506	76180	74752	75118
New case	279	135	69	130
Incidence rate (95% C.I.)	9.22 (8.20–10.37)	17.72 (14.97–20.98)	9.23 (7.29–11.69)	17.31 (14.57–20.55)
Adjusted Hazard ratio (95% C.I.)	Reference	1.664 (1.343–2.061)	Reference	1.839 (1.367–2.473)
Peritoneal dialysis				
Follow up person months	306222	78181	75680	77044
New case	11	16	4	16
Incidence rate (95% C.I.)	0.36 (0.20–0.65)	2.05 (1.25–3.34)	0.53 (0.20–1.41)	2.08 (1.27–3.39)
Adjusted Hazard ratio (95% C.I.)	Reference	4.052 (1.754–9.358)	Reference	3.426 (1.107–10.604)

Abbreviations: C.I.: confidence interval; HD: Hemodialysis; PD: Peritoneal dialysis; VEGF: Vascular endothelial growth factor. ^a^Propensity score matching (PSM) was done by matching: Year of index, Sex, Age at index, Indication, Urbanization, Insured unit type, Marital status, Education level, Co-morbidities (including Hypertension, Diabetes mellitus, Ischemic heart diseases, Hyperlipidemia, Congestive heart failure, and Rheumatic disease). ^b^Incidence rate, per 10000 person-month.

### Incidence rate and adjusted hazard ratios of different indications for anti-VEGF related to dialysis in the age- and sex-matched cohort

**Figure 3:**
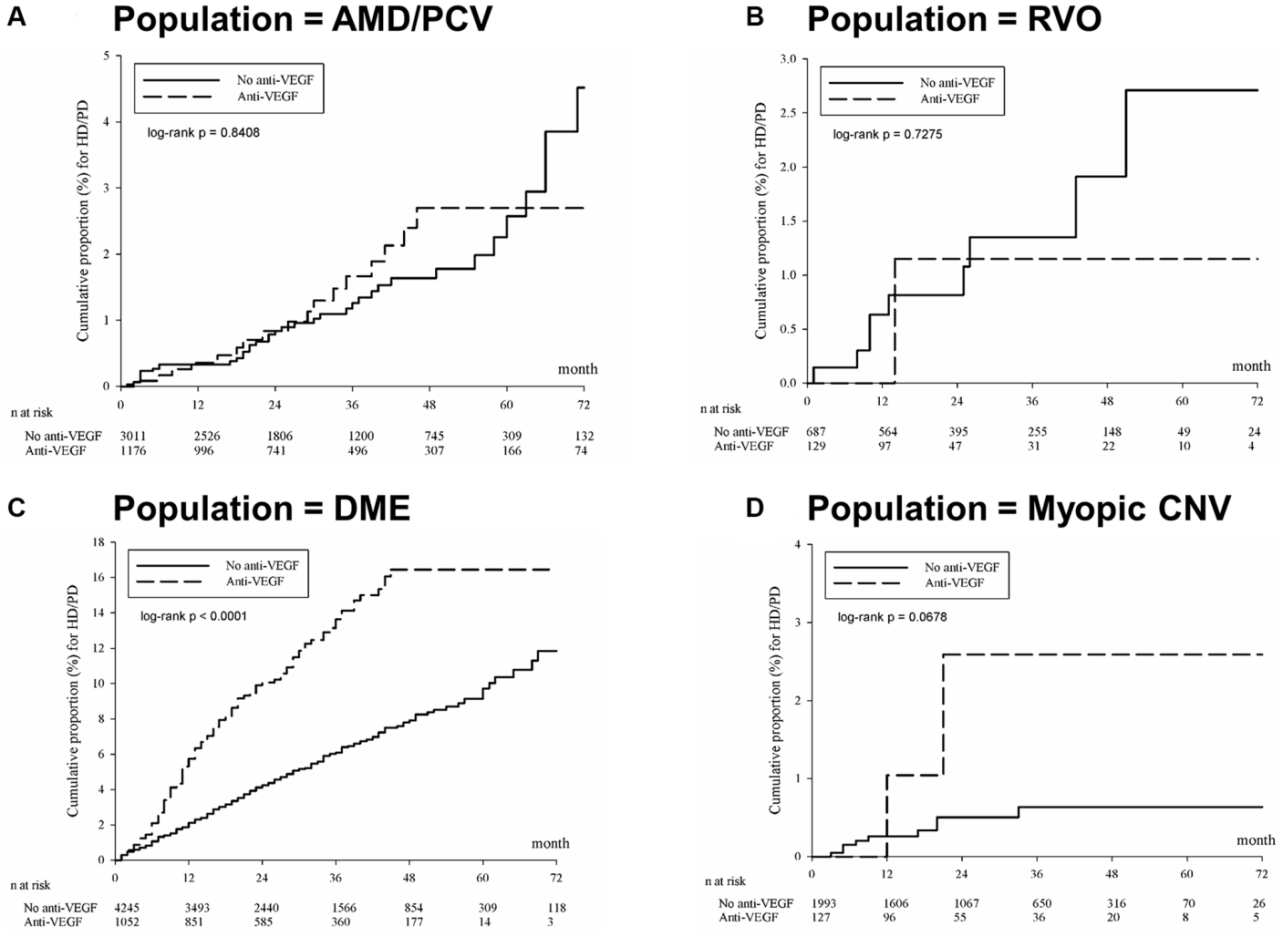
**Kaplan-Meier curves of the cumulative proportion for hemodialysis/peritoneal dialysis in different indications for anti-VEGF treatment.** (**A**) Age-related macular degeneration or polypoidal choroidal vasculopathy. (**B**) Branch retinal vein occlusion or central retinal vein occlusion. (**C**) Diabetic macular edema. (**D**) Myopic CNV. Abbreviations: AMD: age-related macular degeneration; CNV: choroidal neovascularization; DME: diabetic macular edema; HD: hemodialysis; PCV: polypoidal choroidal vasculopathy; PD: peritoneal dialysis; VEGF: vascular endothelial growth factor.

After dividing the study and control groups into subgroups according to their indications (AMD/PCV, RVO, DME, Myopic CNV), subgroup analysis demonstrated that the DME subgroup who received anti-VEGF therapy had a significantly higher incidence rate of becoming dialysis-dependent than their counterparts without receiving the same treatment—39.43 (95% CI: 32.90–47.27) for the anti-VEGF group and 17.45 (95% CI: 15.31–19.89) for the group without anti-VEGF. After adjusting for potential confounders, anti-VEGF injections for DME were associated with a significantly higher risk of becoming dialysis-dependent (adjusted HR: 1.834; 95% CI: 1.448–2.324). The cumulative risk of dialysis was significantly different between the cohorts of DME patients over the entire Kaplan-Meier curve (*p* < 0.0001, log rank test; Figure 3). The incidence rate and adjusted HR of the other subgroups showed no significant differences between the anti-VEGF group and those without anti-VEGF therapy (Table 3).

**Table 3:** Incidence rate of dialysis of the age-and sex-matched population stratifying by different disease indications for intravitreal anti-VEGF treatment.

**Subgroup**	**Incidence rate^a^ (95% C.I.)**		
	**No anti-VEGF *N* = 9936**	**Anti-VEGF *N* = 2484**	**aHR^b^**
Indication			
AMD/PCV	3.93 (2.86–5.40)	4.26 (2.65–6.86)	0.991 (0.544–1.806)
RVO	4.30 (2.24–8.26)	3.08 (0.43–21.90)	0.410 (0.034–4.989)
DME	17.45 (15.31–19.89)	39.43 (32.90–47.27)	1.834 (1.448–2.324)
Myopic CNV	1.61 (0.84–3.10)	5.95 (1.49–23.78)	2.004 (0.303–13.235)

Abbreviations: AMD: Age-related macular degeneration; C.I.: confidence interval; CNV: Choroidal neovascularization; DME: Diabetic macular edema; PCV: Polypoidal choroidal vasculopathy; RVO: Branch retinal vein occlusion or central retinal vein occlusion; VEGF: vascular endothelial growth factor. ^a^Incidence rate, per 10000 person-months. ^b^Adjusted hazard ratio: The covariates including year of index, sex, age, indication, urbanization, insured type, marital status, education level, co-morbidities at baseline.

### Adjusted hazard ratios of different regimens and number of anti-VEGF injections related to dialysis

Two anti-VEGF agents, ranibizumab and aflibercept, that have been eligible for reimbursement by the government since 2011 and 2014, respectively. Our analysis demonstrated that patients who received ranibizumab had a risk of becoming dialysis dependent than the group without anti-VEGF therapy [adjusted hazard ratio: 1.722, (95% CI: 1.388–2.136) in the age- and sex-matched cohort, and 1.896 (95% CI: 1.409–2.551) in the PSM cohort] (Supplementary Table 2). Furthermore, more than 2 injections of ranibizumab were also associated with a risk of becoming dialysis dependent, while only one injection was not associated with risk (Supplementary Table 2). By contrast, aflibercept treatment was not associated with a greater risk of dialysis, nor was any dosage-dependent effect observed.

## DISCUSSION

The present study demonstrated that compared to patients who never received intravitreal anti-VEGF, patients who ever received intravitreal anti-VEGF were associated with 85% higher risk of becoming dialysis-dependent according to the results of group comparison after PSM (Table 2). Given the increasing usage of intravitreal anti-VEGF and the large socioeconomic burden of being dialysis dependent, this minor difference may have a great impact on the current clinical practice. Subgroup analysis showed the risk of becoming dialysis dependent is 83% higher in the patients who received intravitreal anti-VEGFs for DME compared to the patients with DME but did not receive anti-VEGF treatment (Table 3).

Keir et al., [17] demonstrated that intravitreal injection of VEGF inhibits local complement factor H in the rodent kidney, thus inducing alternative complement pathway deposits in the kidney. In previous case reports, intravitreal injections of anti-VEGF agents were noted to impact renal function in patients with either healthy kidneys [20] or with already compromised kidneys [21, 23]. To our knowledge, this large-scale study is the first report to explore the role of intravitreally administered anti-VEGF in the cumulative risk of requiring maintenance dialysis by using a population-level database.

Our study showed that DME patients had a risk of dialysis dependence after the administration of intravitreal anti-VEGF agents (Table 3). Diabetes in the process of renal impairment seemed to be a confounder. The incidence of renal adverse events was reported to be 16–22% in diabetic patients of the Protocol T two-year database [25]. While some articles demonstrated a correlation between intravitreal injection of anti-VEGF agents and impairment of renal function and aggravation of proteinuria in diabetic kidneys [22–24], others showed no such correlation [26–28]. The influence of VEGF-A might be complex for the changes in retinal condition and renal function in diabetic patients, because whereas VEGF-A expression is often upregulated in the kidneys of diabetic rodents, the opposite pattern has been noted in humans [29].

The risk of major systemic adverse events was comparable among different anti-VEGF agents [30]. However, in our study, the patients receiving ranibizumab treatment in the anti-VEGF group had an elevated risk for dialysis-dependent status (adjusted hazard ratio: 1.896; 95% CI: 1.409–2.551), while no statistically significant risk was identified in those receiving aflibercept (adjusted hazard ratio: 1.166; 95% CI: 0.454–2.996) (Supplementary Table 2). Additionally, a slight dose-dependent trend was found in the ranibizumab-administered patients, which showed an elevated risk of being dialysis-dependent after administration of the second dose. Our finding contrasts with the hypothesis based solely on systemic VEGF-A inhibition. Different isoforms of VEGF-A may influence kidney disease development in diabetic patients [29]. Ranibizumab and aflibercept differ in their molecular structures, binding targets and binding affinities to their respective binding sites [31, 32], so theoretically, the risks of causing renal injury may differ from each drug. More clinical and laboratory investigations are required to further elucidate the mechanisms.

Our study results also demonstrated higher risk of PD after the uses of anti-VEGF drugs. The possible explanations of a higher risk of PD after the uses of anti-VEGF drugs when compared to HD might be due to the fact that the patients who were able to attend regular ophthalmologic exams were often with better overall performance and may prefer more autonomy when receiving renal replacement therapy. Hence, the risk of PD appeared higher after the uses of anti-VEGF drugs compared with HD.

For the Kaplan-Meier curves of the cumulative proportion for HD or PD in Figure 2, the survival line of anti-VEGF agents was unchanged after 48 months. The possible explanations might be as follows: The approved doses injections had to be administered within five years following the reimbursement approval under the policy of National Health Insurance reimbursement criteria for ranibizumab and aflibercept during the study period. Moreover, in recent real-world studies, [33, 34] investigators found that most injections were given in the first four years following the initialization of intravitreal anti-VEGF treatment. This may explain the reason why the survival line of anti-VEGF agents was unchanged after 48 months, since further renal adverse effects may not be present after the termination of regular intravitreal anti-VEGF treatment.

Strengths of this study include the large real-world cohort to evaluate possible side effects of anti-VEGF agents on risks of dialysis. Moreover, the similar findings in the age- and sex-matched cohort and PSM cohort re-ascertained our study results. Nonetheless, we do acknowledge some limitations to the presented study. First, the indications, demographic information and comorbidities of the included patients depended on the accuracy of International Classification of Diseases, 9th Revision, Clinical Modification (ICD-9-CM), and International Classification of Diseases, 10th Revision, Clinical Modification (ICD-10-CM) codes, so coding errors might exist and may lead to information bias. Second, due to the strict reimbursement criteria (Table 4) of the National Health Insurance, not all patients with indications of AMD/PCV, DME, RVO or myopic CNV were eligible to receive anti-VEGF treatment. Third, although ranibizumab and aflibercept are both covered by the National Health Insurance, some patients might receive self-paid anti-VEGF treatment, including off-label use of intravitreal bevacizumab. Fourth, DME accounted for over 40% of the indications, the current construction of the propensity score may not protect against potential residual confounding due to diabetes severity, since serial HbA1C is not available in the NHIRD. Fifth, as the primary outcome is receiving dialysis, the baseline renal function is a potential confounder. However, since the dataset of NHIRD did not contain the laboratory data of renal function, we could only adjust the chronic kidney disease in the analyses by relevant ICD codes. Even if our study results indicated a higher risk of developing dialysis in patients receiving intravitreal anti-VEGF agents, more studies, including randomized controlled trials, or new-user design with active comparator or systematic review and meta-analysis, are warranted to clarify the role of anti-VEGF agents in the pathophysiology of dialysis and to replicate our findings.

**Table 4:** The National Health Insurance reimbursement criteria for intravitreal ranibizumab and aflibercept in patients with different indications (released on March 1st, 2020).

	**Initial date of reimbursement**	**Maximum doses/5 yrs**	**Age**	**CRT**	**HbA1c**	**BCVA 20/400-20/40**	**FA**	**OCT**	**ICGA**	**High myopia^a^**
**AMD/PCV**	RBZ: Feb 2011/Dec 2017 AFL: Aug 2014/Nov 2016	7^#^	≧50 yrs (nAMD)			V	V	V	V (PCV)	
**DME**	RBZ: Feb 2013 AFL: Nov 2016	8^#^		≧300 μm	<10%	V	V	V		
**CRVO/BRVO**	RBZ: Jul 2016/Dec 2017 AFL: Nov 2016/Dec 2017	7^#^	≧18 yrs	≧300 μm		V	V	V		
**mCNV**	RBZ: Jul 2016 AFL: Dec 2017	3				V	V	V		V

Abbreviations: AFL: aflibercept; BCVA: best corrected visual acuity; BRVO: branch retinal vein occlusion; CRT: central retinal thickness; CRVO: central retinal vein occlusion; DME: diabetic macular edema; FA: fluorescein angiography; ICGA: indocyanine green angiography; mCNV: myopic choroidal neovascularization; AMD: age-related macular degeneration; OCT: optical coherence tomography; PCV: polypoidal choroidal vasculopathy; RBZ: ranibizumab. ^a^High myopia: myopia over -6.00D and axial length >26 mm.

The National Health Insurance reimbursement criteria of intravitreal ranibizumab and aflibercept in patients with different indications. In patients with a diagnosis of age-related macular degeneration (AMD)/polypoidal choroidal vasculopathy (PCV), the reimbursement criteria include: age 50 years or older; fluorescein angiography (FA), indocyanine green angiography (ICGA) (only for PCV) and optical coherence tomography (OCT) performed within the past month compatible with a diagnosis of neovascular AMD/PCV; and best-corrected visual acuity (BCVA) within 0.05–0.5 (20/400-20/40). In patients with a diagnosis of diabetic macular edema (DME), the reimbursement criteria include: central retinal thickness (CRT) above 300 μm; HbA1c level below 10%; FA, OCT performed within the past month compatible with a diagnosis of DME; and BCVA within 0.05–0.5 (20/400-20/40). In patients with a diagnosis of central retinal vein occlusion (CRVO) or branch retinal vein occlusion (BRVO), the reimbursement criteria include: age 18 years or older; central retinal thickness above 300 μm; FA, OCT performed within the past month compatible with a diagnosis of CRVO or BRVO; and BCVA within 0.05–0.5 (20/400-20/40). In patients with a diagnosis of myopic choroidal neovascularization (mCNV), the reimbursement criteria include: myopia over -6.00D and axial length >26 mm; FA, OCT performed within the past month compatible with a diagnosis of myopic CNV; and BCVA within 0.05–0.5 (20/400-20/40).

^#^Patients with AMD/PCV, DME, CRVO/BRVO, and mCNV receive initial 3, 5, 3, and 3 injections respectively following the first reimbursement approval. Additional 4, 3, and 4 injections for AMD/PCV, DME, and CRVO/BRVO patients will be approved following the second reimbursement. All medication should be injected within five years following reimbursement approval.

In summary, our study demonstrated an increased risk of becoming dialysis-dependent in patients receiving intravitreal anti-VEGF therapy. Furthermore, our data suggest that patients who receive anti-VEGF for DME treatment and those who receive repeated doses of ranibizumab are more susceptible to dialysis treatment. Based on these findings, a regular laboratory workup of the renal function in patients receiving multiple doses of anti-VEGF agents is imperative. Future work is warranted to develop a predictive model to determine the patients at risk of dialysis after anti-VEGF treatment, thereby helping clinicians choose appropriate treatment plans for each individual.

## METHODS

### Data source

The retrospective population-based cohort study was approved by the Chung Shan Medical University Hospital Institutional Review Board (IRB No.: CS1-20108) and by the National Health Insurance Administration. The data extracted from the NHIRD, a database supported by the Taiwan National Health Research Institutes, contain all the medical information of insurance claims from the Taiwanese population. The information was anonymized before release, hence the Chung Shan Medical University Hospital Institutional Review Board obviated the necessity of obtaining participants’ informed consent for this study. We confirmed that all experiments were performed in accordance with relevant guidelines and regulations. The information includes date of birth, sex, place of residence, inpatient and outpatient services, details of medications, intervention procedures, date of admission and discharge, and diagnosis records (based on the ICD-9-CM and ICD-10-CM). In our present study, we used the data of 2 million individuals who were randomly sampled from the NHIRD, namely, the Longitudinal Health Insurance Database (LHID) (from 2000 to 2017). The accuracy of the NHIRD has already been demonstrated by several previous studies [35–37].

The national health insurance program implemented by the government in 1995, now covering 99% of the 23.5 million people residing in Taiwan, has approved the conditional reimbursement of intravitreal ranibizumab and aflibercept (Table 4) since 2011, provided that the application was approved by retinal experts in advance. Furthermore, the switch of anti-VEGF agents after approval was banned by the policy, that is, the patient received the same anti-VEGF throughout the treatment course. In view of the strict criteria of the reimbursement and the large sample size of Taiwan’s NHIRD, it is statistically feasible to assess the incidence of becoming dialysis-dependent status, which is a rare adverse event, among treated patients.

### Study population

Individuals diagnosed with age-related macular degeneration or polypoidal choroidal vasculopathy (ICD-9-CM code 362.16, 362.50, 362.51, 362.52; ICD-10-CM code H35.05x, H35.30, H35.31, H35.32), retinal vessel occlusion (ICD-9-CM code 362.3x; ICD-10-CM code H34.x), diabetic macular edema (ICD-9-CM code 250.5x, 362.0x; ICD-10-CM code E08.3x, E09.3x, E10.3x, E11.3x, E13.3x), myopic choroidal neovascularization (ICD-9-CM code 360.21, 367.1; ICD-10-CM code H44.2x, H52.1x) with three more visits to outpatient services due to previously mentioned diseases during January 2000 to December 2017 were included. For patients who received intravitreal aflibercept (Anatomical Therapeutic Chemical (ATC) drug code: S01LA0) or ranibizumab (ATC drug code: S01LA04), the index date was the day of the first intravitreal anti-VEGF injection. For patients who did not receive intravitreal anti-VEGF treatment, the index date was nested with the paired anti-VEGF patients. All study participants were at risk on the index date. In the present study, we used the time distribution matching method to control immortal time bias [38]. To evaluate the association between intravitreal anti-VEGF and new-onset dialysis, the following exclusion criteria were defined: (1) use of bevacizumab and (2) HD or PD status before the index date. We excluded the patients using bevacizumab because bevacizumab was reimbursed for the indication of cancer treatment in Taiwan. Although systemic uses of aflibercept was approved for cancer treatment, it is not covered by Taiwan's National Health Insurance [39]. Hence, we did not exclude systemic uses of aflibercept in the present study. After the exclusions, 2484 patients who had undergone at least one intravitreal injection of anti-VEGF from January 2011 to July 2017 remained in the study cohort. The study design compared the difference in the risk of new-onset dialysis between anti-VEGF and without anti-VEGF cohorts. Each anti-VEGF patient was assigned to 4 controls matched by age and sex, and 9936 patients without anti-VEGF were included for the control cohort. Patients with and without anti-VEGF therapy were further matched with propensity scores at a 1:1 ratio to minimize selection bias. Propensity scores were calculated using logistic regression to estimate the probability of receiving intravitreal anti-VEGF based on the baseline variables of year of index, sex, age at index, indication, urbanization, insured unit type, marital status, education level, and comorbidities (hypertension, diabetes mellitus, ischemic heart diseases, hyperlipidemia, congestive heart failure, rheumatic disease, all kidney diseases, and specifically chronic kidney disease). After PSM, the groups with and without anti-VEGF therapy were comprised of 2447 patients (Figure 1).

### Main outcome measurement

The primary outcome was new dialysis-dependent status following intravitreal anti-VEGF based on the charge master code for hemodialysis (58001C, 58002C, 58003C, 58007C, 58014C, 58018C, 58019C, 58020C, 58021C, 58022C, 58023C, 58024C, 58025C, 58027C, and 58029C) and peritoneal dialysis (58009B, 58010B, 58012B, 58011C, 58017C, and 58028C) after the index date. To avoid the emergent dialysis due to acute kidney injury, we defined the outcome of new dialysis as at least 2 times of records of dialysis. The study period was from January 1, 2000 to December 31, 2017. The varieties of injected agents and injection times were compared for analyzing their associations with newly performed dialysis. Subgroup analysis, including different indications of AMD/PCV, RVO, DME and myopic CNV, was also performed to identify relationships with newly performed dialysis.

### Identification of comorbidities

Comorbidities were identified to evaluate the health status of each participant and to investigate the correlation between newly needed dialysis and comorbidities. Comorbidities included hypertension, diabetes mellitus, ischemic heart disease, hyperlipidemia, congestive heart failure, rheumatic disease, all kidney diseases, and specifically chronic kidney disease. The comorbidities were identified within 1 year before the index date by relevant ICD codes (Supplementary Table 3).

### Statistical analysis

SAS version 9.4 (SAS Institute Inc., Cary, NC, USA) was used for all analyses. After 1:4 matching with age and sex and PSM, the absolute standardized difference (ASD) was used to evaluate the differences between the study and control groups. An ASD <0.1 indicates a negligible difference between two treatment groups. Chi-square tests or Fisher’s exact tests were used for categorical variables, and Student’s *t*-tests were used to compare continuous variables. Poisson assumptions were used to calculate the incidence rate. Cumulative incidence rates were calculated according to analysis of the cumulative incidence of outcome events. The adjusted HR integrates the patients’ demographic information and comorbidities and was analyzed with multiple Cox proportional hazards regressions. We also conducted subgroup analysis to assess the risk of dialysis-dependent status from intravitreal anti-VEGF treatment, grouped by indications (AMD/PCV, RVO, DME, and myopic CNV), anti-VEGF agents (ranibizumab and aflibercept) and number of injections. Dialysis was also divided into two subgroups: hemodialysis and peritoneal dialysis. The incidence rates of each subgroup were calculated. Statistical significance was defined as *p* < 0.05.

### Ethics approval and consent to participate

The retrospective study was approved by the Chung Shan Medical University Hospital Institutional Review Board (IRB No.: CS1-20108) and by the National Health Insurance Administration. The information was anonymized before release, hence the Chung Shan Medical University Hospital Institutional Review Board obviated the necessity of obtaining participants’ informed consent for this study.

### Consent for publication

The information was anonymized before release, hence the Chung Shan Medical University Hospital Institutional Review Board obviated the necessity of obtaining participants’ informed consent for this study.

### Availability of data and materials

The datasets generated and/or analysed during the current study are available in the Taiwan National Health Insurance Research Database repository, (https://nhird.nhri.org.tw/en/How_to_cite_us.html).

## Supplementary Materials

Supplementary Tables
