# Supplementary material for: Risk of dialysis in patients receiving intravitreal anti–vascular endothelial growth factor treatment: a population-based cohort study
Source: Aging (Albany NY). 2022 Jun 20;14(12):5116–30. doi: 10.18632/aging.204133 (PMC9271293; doi:10.18632/aging.204133)
Supplement: Supplementary Tables [file aging-14-204133-s001.pdf]

## SUPPLEMENTARY TABLES

**Supplementary Table 1. Multiple Cox proportional hazards regression for the estimation of adjusted hazard ratios for hemodialysis or peritoneal dialysis.**

| Variable | aHR[a] (95% C.I.) | |
|---|---|---|
| | Age-and sex-matched | PSM[b] |
| Anti-VEGF injection (ref: Control) | 1.680 (1.358–2.078) | 1.849 (1.378–2.482) |
| Sex | | |
| Male | 1.396 (1.111–1.753) | 1.326 (0.951–1.849) |
| Female | Reference | Reference |
| Age at index | | |
| 20–40 | 1.312 (0.581–2.959) | 1.297 (0.484–3.477) |
| 40–60 | Reference | Reference |
| 60–80 | 0.629 (0.483–0.820) | 0.707 (0.494–1.012) |
| 80–100 | 0.597 (0.407–0.877) | 0.670 (0.361–1.242) |
| Co-morbidities[c] | | |
| Hypertension | 1.832 (1.433–2.342) | 1.791 (1.268–2.531) |
| Diabetes mellitus | 2.696 (1.802–4.033) | 4.062 (1.971–8.373) |
| Ischemic heart diseases | 0.829 (0.587–1.172) | 0.551 (0.304–1.001) |
| Hyperlipidemia | 1.012 (0.831–1.232) | 1.234 (0.930–1.638) |
| Congestive heart failure | 2.238 (1.683–2.977) | 1.651 (1.059–2.575) |
| Rheumatic disease | 1.713 (0.705–4.163) | 0.546 (0.075–3.983) |
| Kidney disease | 3.156 (2.322–4.289) | 3.420 (2.222–5.263) |
| CKD | 2.284 (1.663–3.137) | 1.844 (1.177–2.888) |

Abbreviations: C.I.: confidence interval; CKD: chronic kidney disease; VEGF: vascular endothelial growth factor. [a]Adjusted hazard ratio (aHR): The covariates including year of index, sex, age, indication, urbanization, insured type, marital status, education level, co-morbidities at baseline. [b]Propensity score matching was done by matching: Year of index, Sex, Age at index, Indication, Urbanization, Insured unit type, Marital status, Education level, Co-morbidities (including Hypertension, Diabetes mellitus, Ischemic heart diseases, Hyperlipidemia, Congestive heart failure, and Rheumatic disease). [c]Co-morbidity were identified within 1 year before index date.

**Supplementary Table 2. Multiple Cox proportional hazards regression for the estimation of adjusted hazard ratios for hemodialysis or peritoneal dialysis stratifying by different types of the anti-VEGF agents.**

| Variable | aHR[a] (95% C.I.) | |
|---|---|---|
| | Age-and sex-matched | PSM[b] |
| Model: type of Anti-VEGF | | |
| No anti-VEGF | Reference | Reference |
| Ranibizumab | 1.722 (1.388–2.136) | 1.896 (1.409–2.551) |
| Aflibercept | 1.031 (0.418–2.546) | 1.166 (0.454–2.996) |
| Model: times of ranibizumab | | |
| 0 | Reference | Reference |
| 1 | 1.212 (0.497–2.952) | 1.276 (0.511–3.185) |
| 2 | 2.519 (1.456–4.357) | 2.809 (1.566–5.036) |
| 3 | 1.746 (1.253–2.433) | 1.834 (1.236–2.722) |
| ≥4 | 1.655 (1.266–2.164) | 1.846 (1.321–2.578) |

| | | |
|---|---|---|
| Model: times of aflibercept | | |
| 0 | Reference | Reference |
| 1 | – | – |
| 2 | 1.324 (0.184–9.537) | 1.185 (0.161–8.704) |
| 3 | 1.554 (0.381–6.335) | 1.506 (0.361–6.278) |
| ≥4 | 0.599 (0.147–2.431) | 0.569 (0.138–2.351) |

Abbreviations: C.I.: confidence interval; VEGF: vascular endothelial growth factor. [a]Adjusted hazard ratio: The covariates including year of index, sex, age, indication, urbanization, insured type, marital status, education level, co-morbidities at baseline. [b]Propensity score matching was done by matching: Year of index, Sex, Age at index, Indication, Urbanization, Insured unit type, Marital status, Education level, Co-morbidities (including Hypertension, Diabetes mellitus, Ischemic heart diseases, Hyperlipidemia, Congestive heart failure, and Rheumatic disease).

**Supplementary Table 3. Diagnosis codes for study comorbidities.**

| Diseases | ICD-9-CM codes | ICD-10-CM codes |
|---|---|---|
| Chronic kidney disease | 585 | N18.4, N18.5, N18.6, N18.9 |
| Congestive heart failure | 398.91, 402.01, 402.11, 402.91, 404.01, 404.03, 404.11, 404.13, 404.91, 404.93, 425.4–425.9, 428.x | I50.2x, I50.3x, I50.4x, I50.84, I50.89, I50.9 |
| Diabetes mellitus | 250.x, 277.7 | O24.4, E11.x, E13.x, E88.81 |
| Hyperlipidemia | 272.0, 272.1, 272.2, 272.4, 272.9 | E78.0x, E78.1, E78.2, E78.3, E78.4x, E78.5, E78.70, E78.79, E78.89, E78.9 |
| Hypertension | 401.x-405.x | I10, I11.x, I13.x, I15.x, I16.x, I87.3x, I97.3x, O10.x, O11.x, O13.x, O16.x |
| Ischemic heart disease | 410-414 | I20–I25 |
| Kidney disease | 403.x, 404.x, 582.x, 583.0–583.7, 585.x, 586.x, 588.x, V42.0, V45.1, V56.x | E08.2x, E09.2x, E11.2x, E13.2x, I12.x, I13.1, N03.x, N04.x, N11.x, N18.x, O10.2x, O10.3x, Z49.31, I95.3, T82.43XA, E85.3, R88.0, T82.4x |
| Rheumatic disease | 446.5, 710.0, 710.1, 710.3, 710.4, 714.0–714.2, 714.8, 725.x | M05.1x, M05.2x-M05.9, M31.6, M32.1x, M32.8, M32.9, M33.03, M33.13, M33.2x, M33.90, M33.93, M34.0x, M34.1x, M34.9, M35.3 |
